# Can I catch this ball and do I know if I can? Characterizing the affordance of interceptability for oneself

**DOI:** 10.3389/fpsyg.2024.1397476

**Published:** 2024-05-30

**Authors:** Samruddhi Damle, Reinoud J. Bootsma, Frank T. J. M. Zaal

**Affiliations:** ^1^Department of Human Movement Sciences, University Medical Center Groningen, University of Groningen, Groningen, Netherlands; ^2^Institut des Sciences du Mouvement, Aix-Marseille Université, CNRS, Marseille, France

**Keywords:** affordances, perception-action coupling, manual lateral interception, action boundary, interceptability, perceptual verbal judgments, affordance perception, perceived interceptability

## Abstract

In this study, we aimed to characterize the affordance of interceptability for oneself using a manual lateral interception paradigm. We asked a two-fold research question: (1) What makes a virtual ball interceptable or not? (2) How reliably can individuals perceive this affordance for oneself? We hypothesized that a spatiotemporal boundary would determine the interceptability of a ball, and that individuals would be able to perceive this boundary and make accurate perceptual judgments regarding their own interceptability. To test our hypotheses, we administered a manual lateral interception task to 15 subjects. They were first trained on the task, which was followed by two experimental sessions: *action* and *judging*. In the former, participants were instructed to intercept as many virtual balls as possible using a hand-held slider to control an on-screen paddle. In the latter session, while making interceptions, participants were instructed to call “no” as soon as they perceived a ball to be uninterceptable. Using generalized linear modeling on the data, we found a handful of factors that best characterized the affordance of interceptability. As hypothesized, distance to be covered and ball flight time shaped the boundary between interceptable and uninterceptable balls. Surprisingly, the angle of approach of the ball also co-determined interceptability. Altogether, these variables characterized the actualized interceptability. Secondly, participants accurately perceived their own ability to intercept balls on over 75% of trials, thus supporting our hypothesis on perceived interceptability. Analyses revealed that participants considered this action boundary while making their perceptual judgments. Our results imply that the perceiving and actualizing of interceptability are characterized by a combination of the same set of variables.

## 1 Introduction

In our everyday life, we encounter numerous opportunities to move purposefully through our environment. Whether it is reaching for a coffee cup, climbing a staircase or navigating a busy street, we are accustomed to perceiving possibilities for action. Such action opportunities, or affordances, define our environment (Gibson, 1979; Chemero, 2003; Michaels, 2003; Fajen, 2007; Richardson et al., 2008; Barsingerhorn et al., 2012). Several paradigms have been employed to investigate a myriad of affordances, spanning from braking (Fajen, 2005) and reaching and grasping (Mon-Williams and Bingham, 2011) to sitting (Mark et al., 1990), stair-climbing (Warren, 1984; Mark, 1987), reaching with and without jumping (Ramenzoni et al., 2010; Thomas et al., 2018), and catching (Postma et al., 2018), amongst others.

In this extensive array of research, a predominant focus has been on studying affordances in static conditions. In such static conditions, the properties of the environment remain unchanged over time. For example, consider one of the classics in affordances research, climbability of stairs (*cf.* Warren, 1984; Mark, 1987). Typically, this affordance of climbability is examined on staircases that are fixed, unchanging, still, stable, non-moving and thus static. This in turn means that a staircase that is climbable (one that one can climb) on one instance will remain climbable and will not change over time, unless some properties of the actor change. Or, unless the staircases resemble those from Hogwarts in Harry Potter’s wizarding world. In our muggle world, however, the affordance of climbability does not vary with time.

Yet, often possibilities for action come and go. We encounter everyday situations where some things do change. For example, when deliberating at the curb about crossing a street with approaching cars, the opportunity to cross a gap between the cars without risking contact is affected by a number of factors, such as one’s walking speed, the to-be-crossed-distance, but also the time available to cross and when one starts moving, etc. (Oudejans et al., 1996). Waiting a little longer at the curb does affect the opportunities: they open up and vanish. Thus, this street-crossing affordance is based not only on dynamic properties of the actor, but additionally on the dynamic properties of elements in the environment.

In the present contribution we focus on the counterpart of avoiding contact with moving objects, namely the possibility of making contact with a moving object, as in intercepting a moving ball. A number of studies have addressed such interceptability in the framework of fly-balls, where the to-be-catcher must typically locomote so as to be able to ultimately intercept the ball. For instance, Oudejans et al. (1996) compared running to catch fly balls with judgments of the future side of passing (behind vs. in front of) the observer. The study considered speed- and acceleration-based variables, but was inconclusive with respect to the characterization of the boundary between interceptable and uninterceptable balls. Later studies (Fajen et al., 2011; Postma et al., 2017, 2018) did demonstrate that individual differences in the time needed to cover the distance to the interception location of the balls was a significant factor in where this boundary would be. Finally, Postma et al. (2022) showed that the time needed for an individual to cover a certain distance is co-determined by the maximum speed and the maximum acceleration that they can reach.

One reason to study the interceptability of fly balls is the endeavor to give affordances their role in the control of action (e.g., Postma, 2019). Fajen and colleagues have argued that extant accounts for the control of action are incompatible with the notion that perception is of affordances (e.g., Fajen, 2007). With respect to running to catch fly balls, when considering the forward-backward component of the movement, the dominant account for the visual control is optical-acceleration cancellation (Michaels and Oudejans, 1992; McLeod and Dienes, 1996). However, whereas optical acceleration seems to be used in the control, it is not used in the perception of the (im)possibility of successful control (i.e., [un]interceptability; see Postma et al., 2017, 2018). Identifying the information for the affordance of interceptability, therefore, might be an entry into a true affordance-based account of this type of interception (*cf.* Postma, 2019). Whereas progress toward an affordance-based account of the visual control of interception has been made studying the running to catch fly balls, having a second paradigm for developing such account seems helpful.

In the present study, we addressed interceptability in the framework of lateral manual interception. This interception task that has been studied extensively (Peper et al., 1994; Montagne et al., 1999, 2000; Dessing et al., 2005; Jacobs and Michaels, 2006; Michaels et al., 2006; Arzamarski et al., 2007; Ledouit et al., 2013). For the present purposes we adopted a virtual lateral manual interception paradigm, in which participants manually control a virtual paddle that can move laterally across the bottom side of a large computer screen. The task is to use the paddle to intercept virtual balls that move from the top of the screen to the bottom. We used uniformly moving balls that could arrive at different distances from the paddle starting position, following trajectories that could have different angles with respect to the vertical. Rather than including only potentially interceptable balls, as done in all previous studies of manual lateral interception, in the present experiment we administered both interceptable and designed-to-be-uninterceptable balls to the participants.

The present study intends to be a first step in the characterization of the affordance of interceptability in manual interception. The study was modeled after the fly-ball studies by Postma et al. (2014, 2017, 2018), now using a paradigm with full control of ball kinematics. In this contribution, we aim to address two questions: First, what makes a ball interceptable or not? In a manual lateral interception paradigm, which factors characterize the affordance of interceptability of a ball? Second, how reliably can people perceive this affordance for themselves? Participants performed a manual lateral interception task with a range of interceptable and uninterceptable balls. Using a hand-held slider coupled to the on-screen paddle, they were tasked with intercepting balls that moved down the screen at varied constant speeds (*action* task). In a *judging* task, they were instructed to make verbal judgment calls (“no”) when they perceived a ball to be uninterceptable. They were allowed to move their paddle while making judgments, such that the choice and timing of abandoning their movement and calling “no” was left up to the participants.

## 2 Materials and methods

### 2.1 Participants

For this exploratory study, we recruited 15 right-handed young adults (9 females, 6 males) from the University of Groningen, with an average age of 24.5 ± 2.87 years (M ± SD). All provided written consent before participating in our study. The inclusion criteria for participants were normal or corrected-to-normal vision and no reported or apparent physical injuries or disabilities.

The experiment was approved by the Ethics Board of the UMCG (University Medical Center Groningen, University of Groningen, Netherlands). The protocol was in accordance with the Declaration of Helsinki.

### 2.2 Experimental set-up

**Figure 1:**
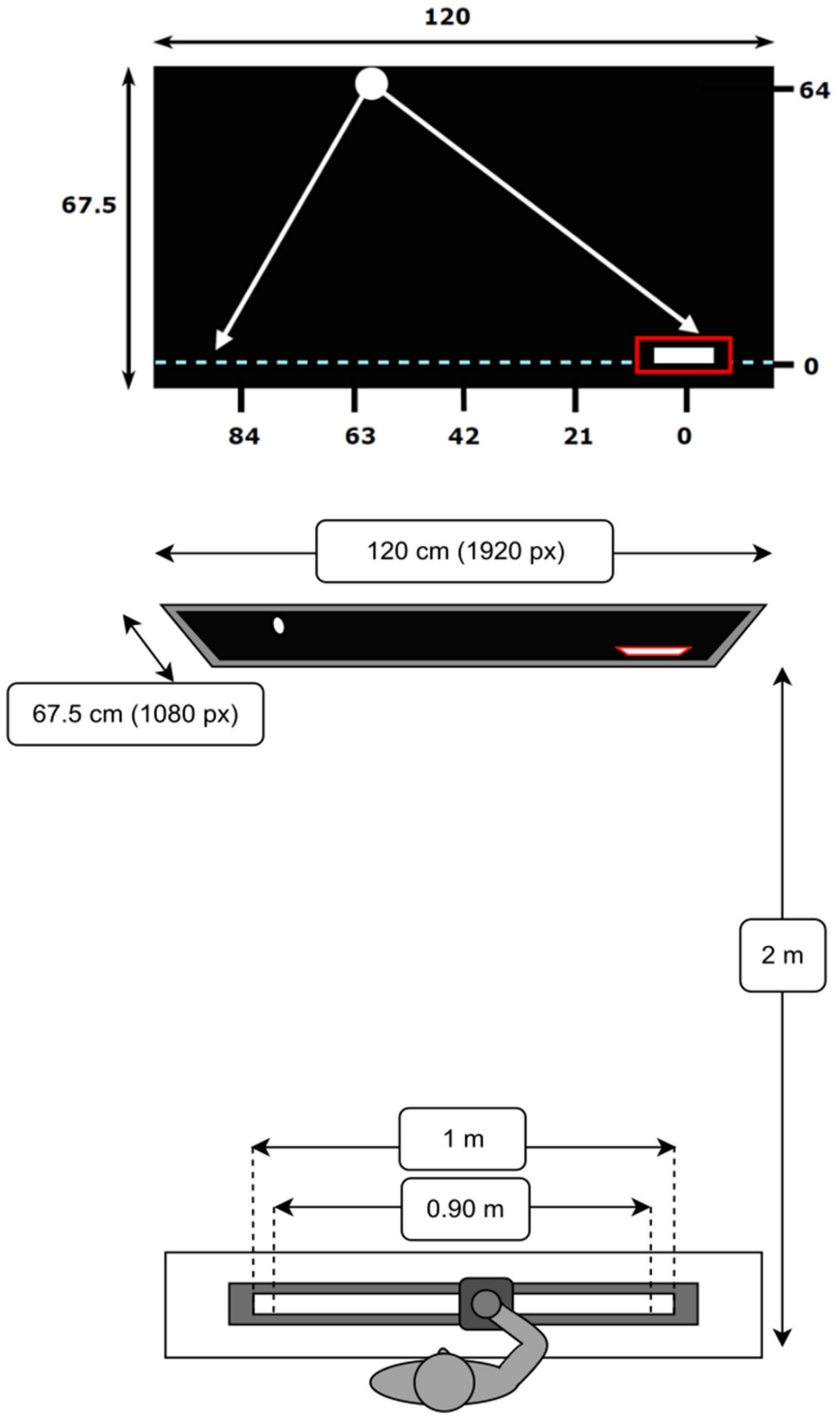
Visual representation of the screen and task dimensions in centimeters (not to scale). The fixed starting position is indicated by the red rectangle at the bottom right position on the interception axis (0,0). The five ball arrival positions are as displayed above. The axis is initialized to 0 on the right-side and in increasing order to the left so as to align with increasing distance from start. Schematic top view of the slider and TV screen.

This study adopted a lateral manual interception paradigm. The experiment was conducted in a darkened room without windows. Participants were seated on a chair at the center of a table, facing a large TV screen of dimensions 120 × 67.5 cm (Samsung 55” QLED QN95A, with a resolution of 1920 × 1080 pixels). We used a HP computer (Windows 11) to run the experiment, which was designed using PsychoPy®, an open access python-based software for designing and conducting experiments (Peirce et al., 2019). Seated at a distance of 2 m, participants viewed the center of the screen at eye height (Figure 1).

Participants were instructed to use their right hand to laterally move a hand-held knob mounted on a slider that was coupled to an on-screen paddle (4.5 cm wide, 0.8 cm high). The objective was to intercept virtual balls (2-cm diameter circles) which moved down the screen at constant speed using the paddle. The slider, which moved over a rail, could cover a 1-m distance. It could be smoothly displaced between both extremities. The slider-paddle system was calibrated such that extreme end positions on screen could be reached by the paddle without physically moving the slider to the extremities, that is, 90% of the slider range corresponded to 100% of the on-screen paddle range. The slider-rail device was constructed in-house using a linear potentiometer and worked like a linear-positioning device, operating at a sample frequency of 100 Hz.

The virtual paddle could move laterally across the full 120-cm (1920 pixels) length of the invisible interception axis, located 2 cm from the bottom of the screen (see Figure 1). The balls moved down the screen, at constant speeds, from beyond the top of the screen, such that they appeared to move smoothly at the start of each trial, covering a vertical Y distance of 64 cm (1024 pixels). Unless otherwise specified, the positions and distances reported from here on correspond to distances on the screen, with the origin corresponding to the paddle starting position on the interception axis (see Figure 1). A Movo M1 USB Lavalier microphone was used to record participants’ verbal judgments of saying “no” (*judging* session, details below).

The virtual positions of the paddle and ball were sampled at a frequency of 100 Hz using the PsychoPy® software. This software was also used to implement the experiment with customized python code segments. The recorded data was stored on a secured research drive within the environment of the UMCG network.

### 2.3 Design and procedure

The experiment comprised three sessions, conducted consecutively and interspersed with 10-min breaks. Participants were instructed to intercept white balls that moved down a black screen at various speeds, trajectories and angles using the white paddle to bounce the ball back up.

Prior to each trial, participants were required to move their paddle to a fixed starting position on the right side of the interception axis (positioned at *X* = 0 cm), marked by a red box (5.5 cm wide, 1 cm high, see Figure 1). If the entire paddle stayed inside the box for a predetermined duration (1 s), the box disappeared and the trial began with a ball moving down the screen. Contact of the ball with the paddle resulted in a successful interception, signaled by the ball bouncing back upwards (paddle turned green). If a ball was not intercepted successfully, the ball continued its trajectory downward beyond the interception axis and was considered a miss (paddle turned red). On average, a trial lasted approximately 8 s in total. At the end of each trial, the box reappeared and participants had 10 s to return the paddle to the start position.

In all three sessions, the ball trajectory conditions remained identical. Five lateral ball departure positions (BDP) at *Y* = 64 cm were combined with five lateral ball arrival positions (BAP) at *Y* = 0 cm on the interception axis, resulting in 25 unique rectilinear ball trajectories. These five positions each were constructed at equal intervals, *X* = 84 cm, 63 cm, 42 cm, 21 cm, 0 cm (with 0 cm being the paddle start position on the right side of the interception axis, see Figure 1). On each trial a random offset of a magnitude between −10.5 and + 10.5 cm was added to both the selected BDP and BAP, resulting in a lateral shift of the entire trajectory while retaining its orientation. Balls could thus appear at lateral position ranging from *X* = 94.5 cm to *X* = −10.5 cm, covering 95% of the interception axis. Further, we manipulated the vertical ball speeds as a function of the duration it took the ball to cover the distance from the top to bottom of the screen. Ball flight times ranged from 1.6 s to 0.6 s, varying across the sessions.

Prior to the experimental sessions, each participant provided a measure of their fastest movement speed. Starting on the right (paddle start position at *X* = 0 cm), they used the paddle to make rapid (ballistic) movements to hit a stationary ball positioned on the left side of the interception axis (*X* = 92 cm, *Y* = 0 cm). This measure was meant to provide an indicator of individual action capabilities of every participant in terms of the least duration they required to cover this fixed distance. This was labeled the minimum movement time (MT).

The first session (*training*) served as familiarization and provided an estimate of participants’ performance on the task. In this session, the 25 ball trajectories were combined with two vertical ball speeds associated with ball flight durations of 1.6 s and 1.2 s, with ball motion conditions presented in a randomized order. Participants performed three blocks of 50 trials, for a total of 150 trials. Each trajectory from a particular BDP to a particular BAP (with the random offset) for a specific ball flight duration was presented thrice within this session.

The next two sessions were labeled as a*ction* session and *judging* session. Their order was counterbalanced across all participants, such that half of them did the *action* session first, followed by *judging*, and vice versa for the remaining participants. Both sessions were similar except for two crucial differences; the instructions and task objective. In the *action* session, participants were instructed to “intercept as many balls as possible” and to maximize their interception score. In the *judging* session, on the other hand, participants were instructed to call “no” as soon as they perceived a ball to be uninterceptable. They could choose whether to abandon their interceptive action or keep moving after they had made their verbal judgment. It was highlighted that the task focus was on the (timing of the) judgment, and not on maximizing the interception score, thus implying that it was acceptable to miss balls during this session. In both these sessions, the 25 trajectories were combined with three vertical ball speeds associated with ball flight durations of 1.2 s, 0.8 s, and 0.6 s. Participants performed three blocks of 75 trials for a total of 225 trials per session. Each trajectory from a particular BDP to a particular BAP (with the random offset) for a specific ball flight duration was presented thrice per session.

### 2.4 Data acquisition and analysis

Success rate was calculated as the total percentage of balls intercepted in each session. A trial was classified as a successful interception if the ball bounced back up after making contact with the paddle.

In the *judging* session, verbal judgments recorded through the microphone were saved as separate wav files for each trial. Audacity®, an audio analysis software (version 3.4.2), was used to acquire timestamps of the “no”-calls. For the timing of calling “no”, we synchronized the time series of all trials such that they were initialized to *t* = 0 s at the moment the ball began to move.

### 2.5 Statistical analysis

For the statistical analyses, we used Generalized Linear Mixed Effects Regression (GLMER) analysis to examine which variables were related to interceptability. The GLMER is a special type of regression that can account for nested dependencies which tend to occur in repeated measures designs. This is also the case with the present study, since all participants were administered all the experimental conditions, giving rise to nested dependencies due to multiple measurements. The GLMER analysis allows the variation in performance to be attributed to relevant empirically controlled factors such as ball arrival positions and vertical ball speeds (fixed effects), but also in part to the individual variation that came with each participant who repeated all the sessions (random effects). It thus enables dissociating the contributions of these separate components which are interdependent (for further details on mixed models, see: Tagliamonte and Baayen, 2012; Winter, 2013; Winter and Wieling, 2016).

**Figure 2:**
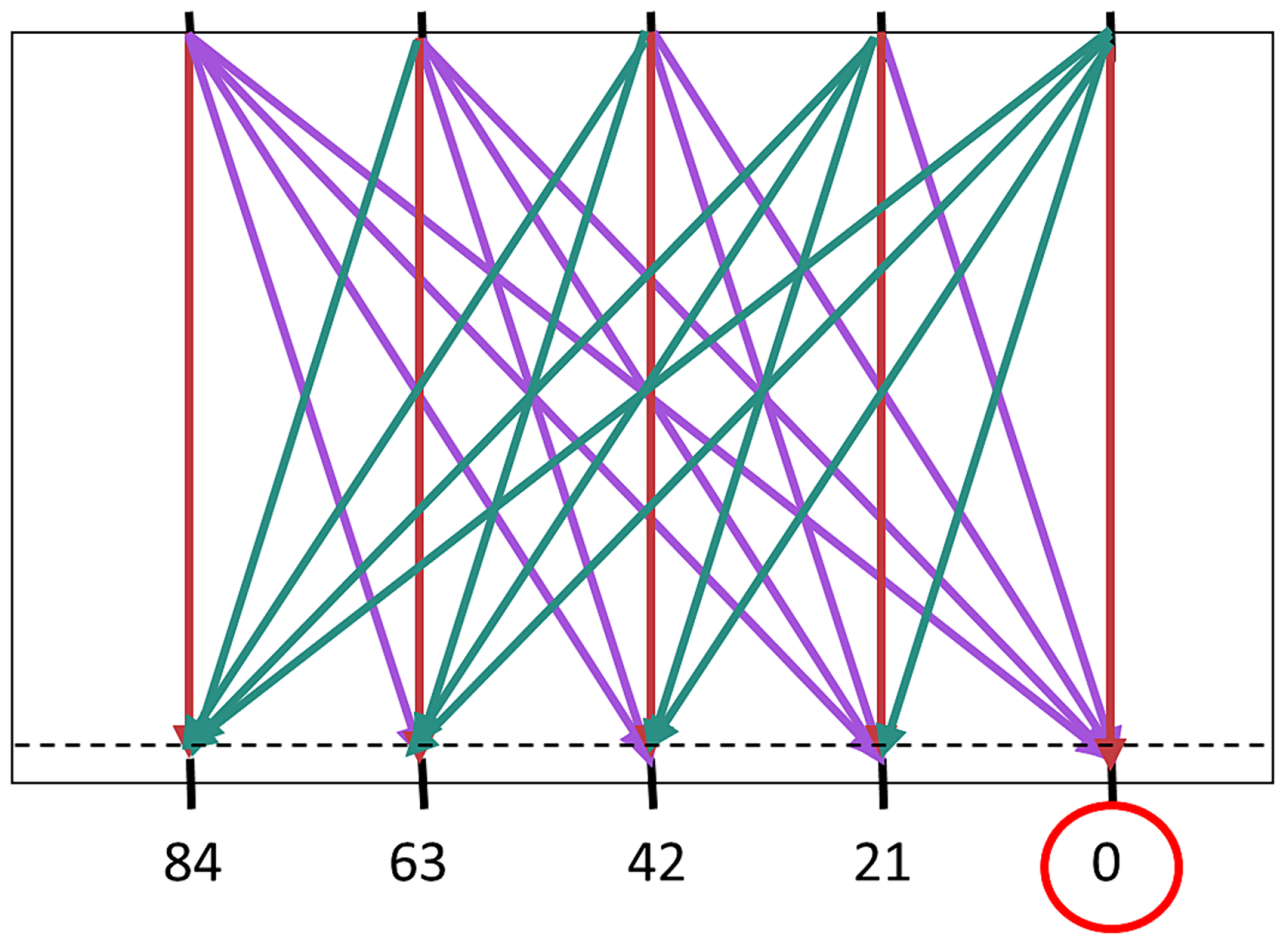
Angle of approach (AoA) defined by the angle the approaching ball makes with the vertical. The absolute angles of approach (Z-scores in brackets) were as follows: 0° (0), 17.28° (0.59), 31.89° (1.09). 43.02° (1.47), and 51.21° (1.76). Balls moving from the top right to the bottom left are classified with a positive AoA (cyan), whereas those moving from top left to bottom right have a negative AoA (purple).

For the GLMER model, variables of ball arrival position (BAP) and ball departure position (BDP) were transformed into continuous predictors of distance from start (D) and the angle of approach (AoA). The distance from start (D) was calculated as the distance from the paddle starting position to the ball arrival position. The angle of approach (AoA) was operationalized as the angle (in degrees) under which the ball approached the horizontal interception axis (depicted in Figure 2). Balls moving from the top right to the bottom left were classified with a positive AoA, whilst those moving from the top left to bottom right were classified with a negative AoA. The balls that moved perpendicular to the interception axis (vertical trajectories) had an AoA of zero. The ball flight time (T) was considered as a factor with comparisons at different levels (further details in the Results section).

The GLMER model used the logit link function as the distribution was binomial (dichotomous outcome variable). The continuous variables (or predictors) were converted to Z-scores for the purpose of the analyses. This enabled variables of different orders of magnitude to be scaled and analyzed as per the prerequisites of the GLMER analysis. All the continuous predictors were centered. We began with an intercept-only model. Using a step-wise forward approach, we added other predictors to the model, and retained only those which led to a decrease of the *Akaike Information Criterion* (AIC) by 2 or more. Predictors that correlated highly with each other were not included in the model, to avoid multicollinearity. We performed this procedure until the model could not significantly improve any further. The entire outputs and specifications of each model are available in the Supplementary material.

## 3 Results

### 3.1 Training session

Table 1 presents the success rates of the individual participants in the *training* session (with ball flight times of 1.6 and 1.2 s). On average, success rates were 91.60%. Success rates improved over the blocks of trials (*M* = 87.47, 93.07, and 94.40%, for Blocks 1, 2, and 3, respectively). We performed GLMER analysis on interceptability as a function of Block (see Supplementary Table S1 for the final model). Block was a significant predictor and post-hoc tests showed significant differences in interceptability between Block 1 and Block 2 (*p* < 0.001) and between Block 1 and Block 3 (*p* < 0.001) but no significant difference between Block 2 and Block 3 (*p* = 0.28).

**Table 1:** Interception success rates for individual participants per session, along with their minimum movement time (MT) in seconds and the frequency of “no”-calls given during the *judging* session.

Participant	Gender	Training session (%)	Action session (%)	Judging session (%)	Min. Mvmt. Time (s)	Freq. “no”-calls
S1	F	87.33	53.33	52.45	0.66	26
S2	F	87.33	62.67	61.33	0.37	36
S3	F	96.00	67.55	59.56	0.34	50
S4	M	92.67	78.67	80.45	0.24	25
S5	F	91.33	63.56	63.56	0.44	30
S6	F	92.00	67.11	57.78	0.47	53
S7	F	93.33	75.11	73.78	0.52	6
S8	M	96.67	71.56	68.45	0.42	28
S9	F	87.33	62.22	55.56	0.29	31
S10	M	90.00	61.33	50.22	0.30	60
S11	M	97.33	72.89	64.89	0.39	25
S12	F	83.33	52.00	42.67	0.42	96
S13	M	92.00	68.00	62.22	0.40	55
S14	M	94.67	70.22	71.11	0.46	29
S15	F	93.33	68.00	72.00	0.37	17
Mean		91.64	66.28	62.4	0.41	37.80
SD		5.69	7.94	10.67	0.10	21.96

### 3.2 Action session

In the *action* session, with ball flight times of 1.2, 0.8 and 0.6 s, participants were instructed to intercept as many balls as possible. The individual success rates can be found in Table 1.

**Figure 3:**
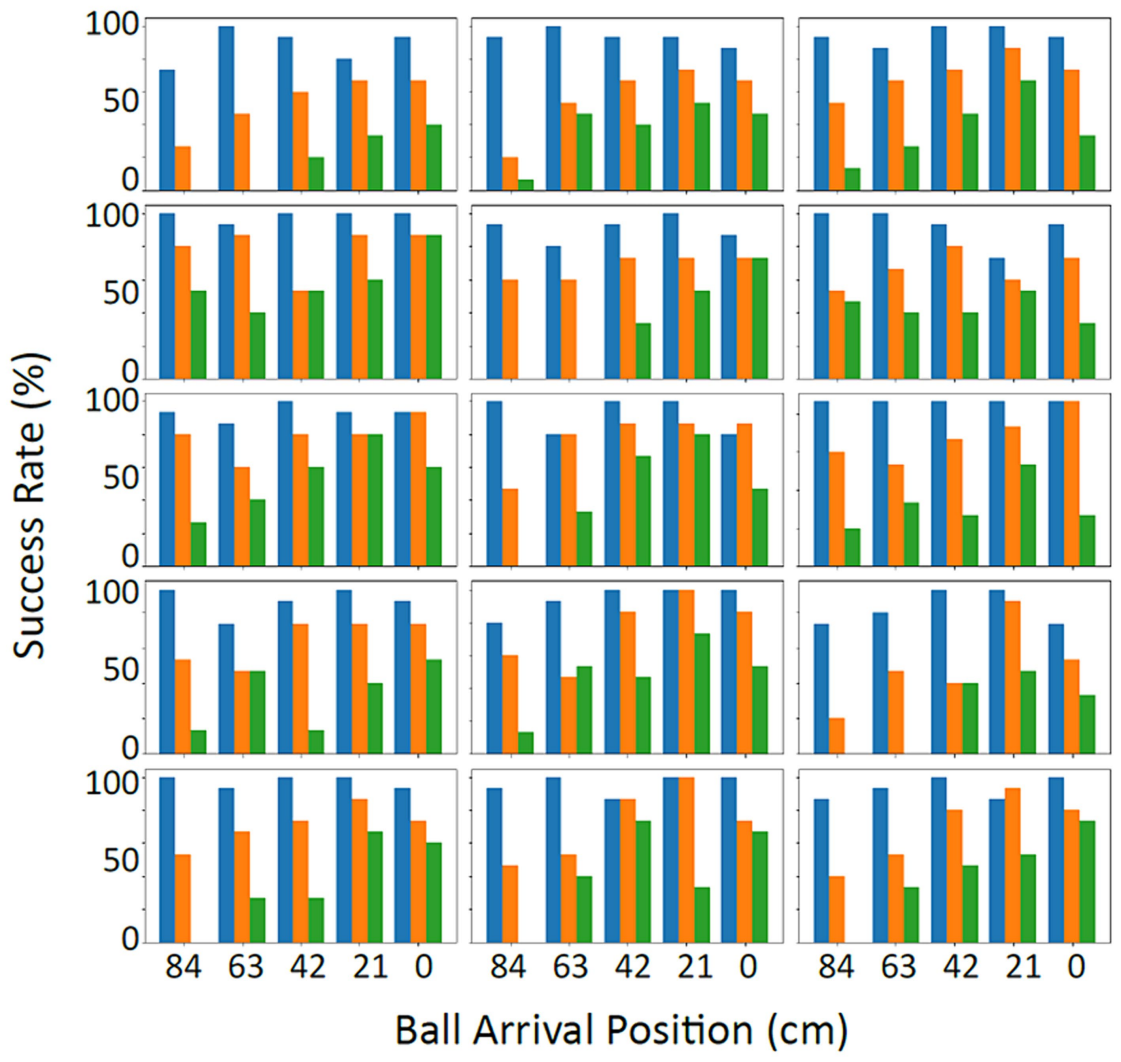
Success rates in percentage per participant (Action session) as a function of ball arrival position (BAP) and ball flight time. The three ball flight times, respectively, were 1.2 s (slow – blue), 0.8 s (intermediate – orange) and 0.6 s (fast – green). The starting position was at the right-most BAP (0 cm).

As expected, several factors did affect the performance. As an illustration, Figure 3 presents the success rates of the individual participants, here as a function of binned ball arrival position (BAP) and ball flight time. Two observations can be made. First, in line with our predictions, when participants did not have enough time to cover the distance to the location at which the ball passed the interception axis (i.e., for balls arriving more to the left side of the screen at the higher vertical speeds), success rates declined. As can be seen from Figure 3, the location of this boundary between interceptable and uninterceptable balls differed across individuals. Second, the vertical speed of the balls also seemed to have an additional effect on success rates even for balls that were clearly within reach. For instance, when we consider the balls that passed the interception axis close to where the participants started their movement (i.e., close to *X* = 0 cm), success rates decreased with shorter ball flight times.

To formally assess the contributions of the various variables to the probability of success in the task, we used Generalized Linear Mixed Effects Regression (GLMER). We considered the factors of distance to the interception location (D: the distance from the paddle starting position to the ball arrival position), ball flight time (T), angle of approach (AoA), and minimum time for participants to cover full screen width (MT) as fixed effects^1^, and we included participant (P) as random intercept.

As can be seen in Table 2 (Interceptability; see also Supplementary Table S2), the final model included all of the aforementioned factors except the minimum time to cover the full screen width (MT). As hypothesized, we observed a significant influence of distance to the interception location (D) as well as ball flight time (T) on interceptability. Unsurprisingly, as the distance to be covered to the interception location increased, the probability of the ball being intercepted decreased. Analogously, the shorter the ball flight time (i.e., higher the vertical ball speed), the lower the probability of interception and vice-a-versa.

**Table 2:** Summary table of all final GLMER models with the estimates and standard errors for each factor included in the model.

Factor-wise estimates (b) and standard errors of each GLMER model			
GLMER model	Interceptability	Verbal Judgment	Congruency
Intercept	1.23 ± 0.13	−3.69 ± 0.33	2.81 ± 0.19
D	−0.66 ± 0.07	1.70 ± 0.11	−1.25 ± 0.12
T1	1.39 ± 0.10	−2.36 ± 0.15	1.62 ± 0.15
T2	3.15 ± 0.13	−4.98 ± 0.29	4.02 ± 0.36
AoA	0.29 ± 0.06	−0.51 ± 0.09	0.52 ± 0.08
D x T1	0.26 ± 0.10	X	−1.28 ± 0.14
D x T2	0.67 ± 0.13	X	−0.82 ± 0.30
D x AoA	−0.39 ± 0.05	0.63 ± 0.07	−0.21 ± 0.06
Equation	Interceptability ~ D + T + AoA + D x T + D x AoA + (1 | P)	Verbal Judgment ~ D + T + AoA + D x AoA + (1 | P)	Congruency ~ D + T + AoA + D x T + D x AoA + (1 | P)

The three models are on Interceptability (*action* session), Verbal Judgment (*judging* session) and Congruency (between verbal judgments and model predictions) respectively. An ‘X’ denotes the absence of a significant predictor in the respective model. D: distance from start; T: ball flight time comparisons at two levels (T1: 0.8 s – 0.6 s and T2: 1.2 s – 0.6 s) and AoA: angle of approach.

**Figure 4:**
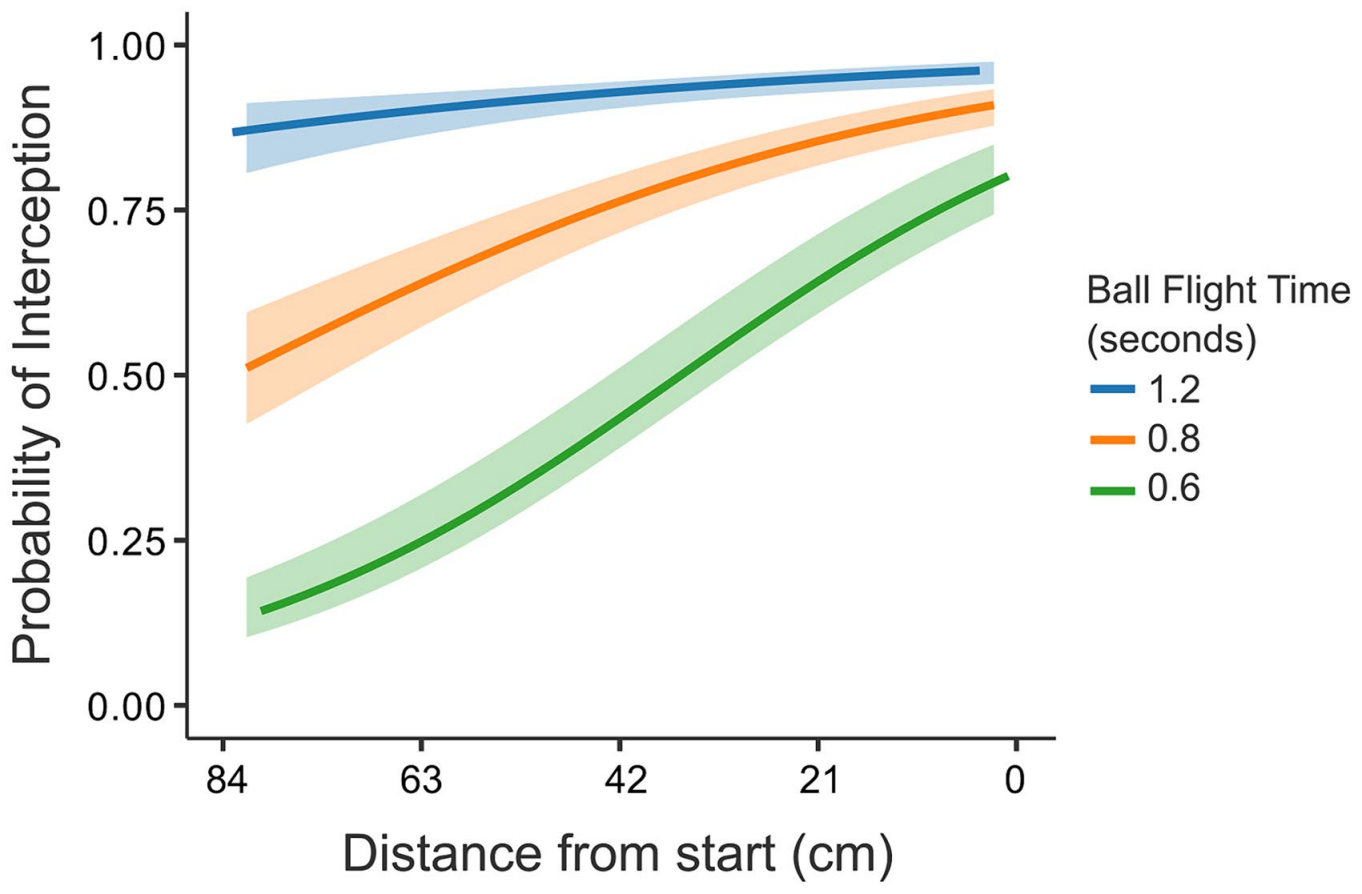
Interaction effect D × T on probability of interception. The three ball flight times, respectively, were 1.2 s (slow - blue), 0.8 s (intermediate – orange) and 0.6 s (fast – green). The shaded region around the curves represents the 95% confidence interval. The *X*-axis represents the distance to be covered from start. The starting position was on the right side of the *X*-axis (0 cm).

The interaction of the factors of D and T also codetermined the probability of a ball being intercepted (see Figure 4). The factor of ball flight time seemed to have played out in two ways. First, as hypothesized, ball flight time had a strong effect for balls that ended up passing the interception axis most to the left of the screen. As the ball flight time determined the time available for the paddle to reach the location where the ball would pass the interception axis, at longer distances to be covered, the time for covering this distance was simply too short. This might be illustrated, for instance, in some of the individual participants in Figure 3, where a clear effect of ball flight time on success rates can be seen for the ball arrival positions most to the left. Second, and not anticipated, the ball flight time appeared to affect participants’ accuracy. This was seen in cases where the distance to be covered was small (right-side of the screen), where the balls with shorter flight times were still missed more often (for an illustration, see the effects of ball flight time on the success rates of the individual participants for the most right-sided ball arrival positions in Figure 3).

**Figure 5:**
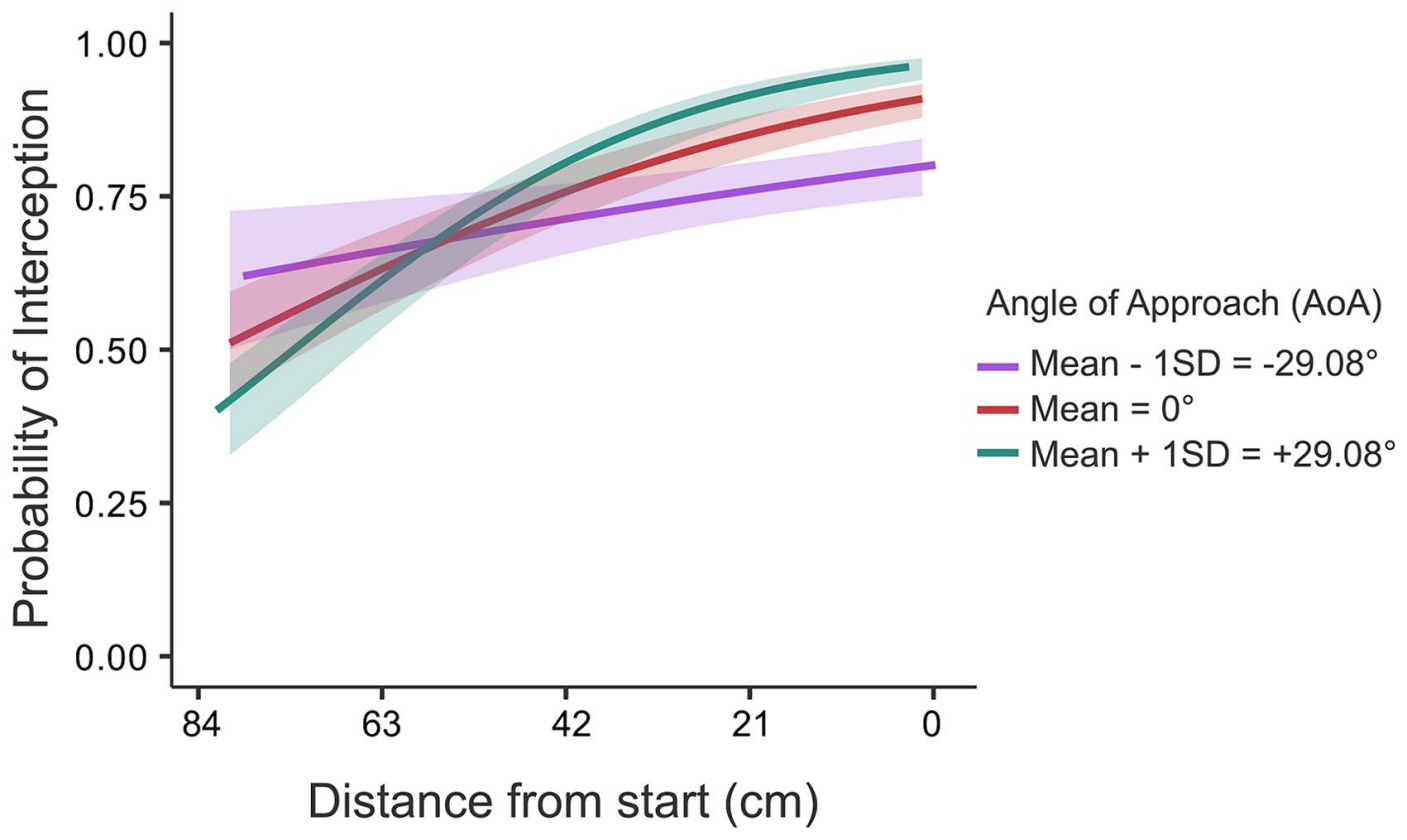
Interaction effect of D x AoA on probability of interception. The three curves represent the angles of approach respectively: negative (purple), zero (red) and positive (cyan). The shaded region around the curves represents the 95% confidence interval. The *X*-axis represents the distance to be covered from start. The starting position was on the right side of the *X*-axis (0 cm).

Finally, the third variable that contributed to the final model was the angle of approach (AoA), defined as the angle under which the ball approached the interception axis (with positive angles for balls moving from top right to bottom left; see Figure 2). The contribution of the AoA to the model was a somewhat surprising finding, both in its direct relation to the outcome, and in the interaction effect with distance to the interception location. The interaction effect is shown in Figure 5.

Two observations can be made from Figure 5. First, as discussed before, overall, the probability of interception decreased as the distance to be covered increased, irrespective of AoA. Second, and more interestingly, there was a cross-over effect on probability of interception as a function of AoA. For the balls that moved with a positive AoA (i.e., moving leftward while moving down), up until about two-thirds of the screen width the probability of interception was higher than when the balls moved down under a negative AoA (i.e., moving rightward while moving down). Beyond about two-thirds of the screen width, the effect of the angle of approach was reversed. In other words, for the majority of balls (the balls that ended up at the right two-thirds of the interception axis), interceptability (i.e., probability of interception) was higher for balls that moved away from the starting location, along the main direction of paddle movement, than for balls that moved horizontally toward the starting position, counter to the main direction of paddle movement.

### 3.3 Judging session

This session comprised comparable trials to the *action* session but with altered instructions; ‘Call “no” as soon as a ball is perceived to be uninterceptable’. Participants had the choice of continuing to try to make the interception or abandon their movement after calling “no.” Two trials were excluded from analyses as the “no” was called before the ball started falling, i.e., in the absence of any visual information about ball movement. The analyses henceforth refer to the remaining trials (*n* = 3373). The success rate on the interception task in this session, 62.40 ± 10.67%, was similar to that of the *action* session (see Table 1).

**Figure 6:**
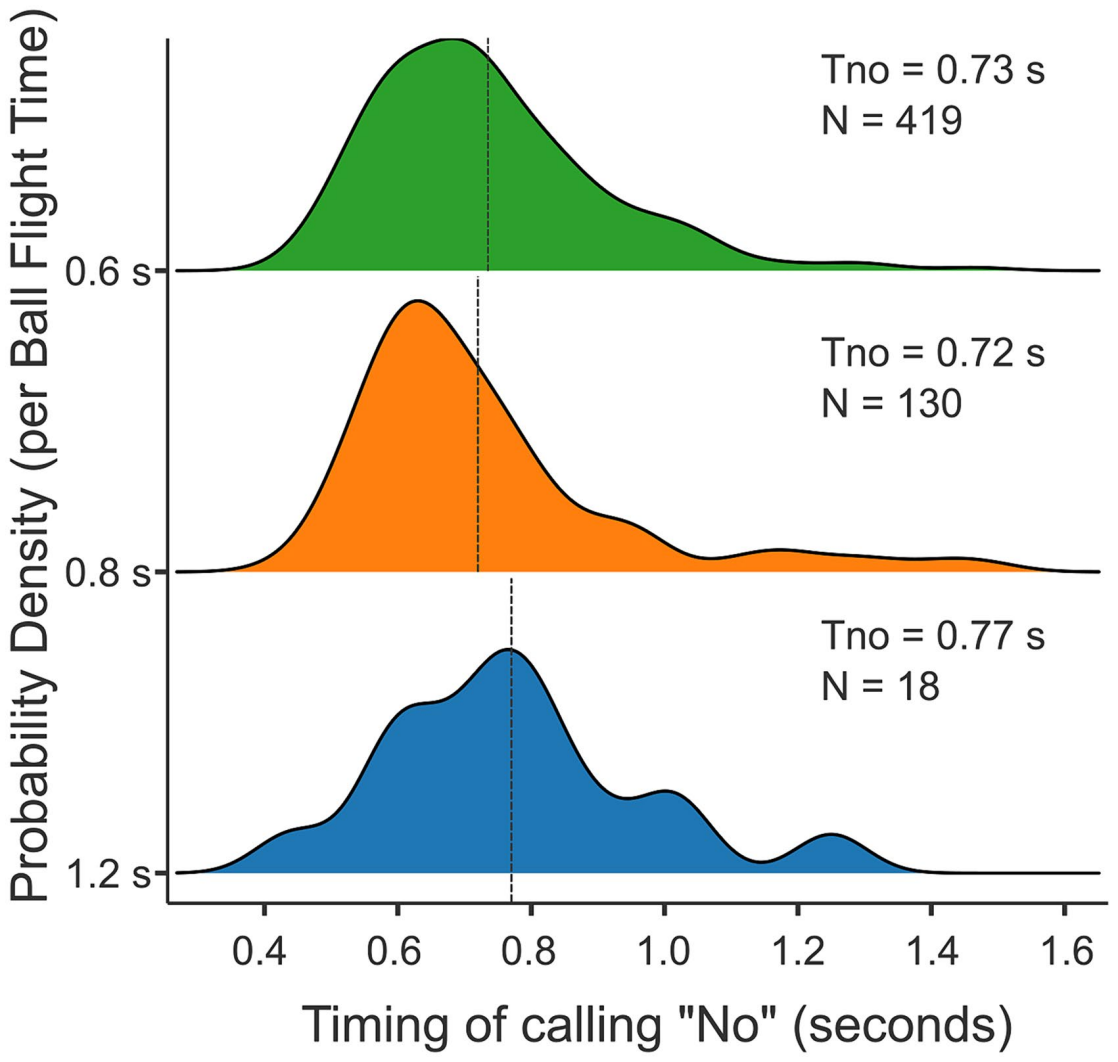
Probability density plots per ball flight time (in seconds) relative to the timing of calling “no” (in seconds). The mean timing of calling “no” (Tno) is 0.73 s, 0.72 s and 0.77 s per ball flight time for the fastest (0.6 s), intermediate (0.8 s) and slowest (1.2 s) conditions, respectively. The frequency of “no”-calls (N) per condition is 419, 130, and 18, respectively.

From the total set of trials, a verbal judgment was made on only 567 trials, which accounts for 16.8% of trials. On average, participants took 0.730 s to indicate that a ball was uninterceptable by calling “no” (SD = 0.185 s). Figure 6 gives the distributions of the times taken to call “no”-s for the three ball flight times separately. It seemed that participants took somewhat longer at the longest ball flight time (*T* = 1.2 s: *M* = 0.774 s, SD = 0.333 s, *N* = 18) than for the two other ball flight times (*T* = 0.8 s: *M* = 0.728 s, SD = 0.206 s, *N* = 130; *T* = 0.6 s: *M* = 0.731 s, SD = 0.169 s, *N* = 419). As can be seen in Figure 6, almost all “no”-calls came before the balls passed the interception axis for the balls with the longest flight time (1.2 s); “no”-calls, on average, came before the balls passed the interception axis for the intermediate ball flight time (0.8 s), and a substantial number of “no”-calls came after the balls passed the interception axis for the shortest ball flight time (0.6 s). However, when we assume a time delay of 200 ms between realizing that a ball will not be interceptable and a voiced “no,” only a minority of these calls would qualify as after the event. In the analyses to be presented, we retained all trials.

Table 3 shows the frequency of calling “no” relative to the outcome of the trial. This table shows that in over 75% of the trials, the calls were in line with the actual outcome of the trials. Perhaps most surprising are the more than 20% of the trials in which the balls were missed and still a call was not made. We will further on return to these trials (or, more precisely, to the similar situation in which we would have predicted that the ball would be uninterceptable and still a call was not made).

**Table 3:** Classification table of *judging* session with respect to actual interception performance result (interception or no interception of the ball) and frequency of verbal calls being made (“no”-call present) or not (“no”-call absent).

		Performance result		Total
		Interception	No interception	
Verbal Judgment	Call made (“no”-call present)	53	514	567
		**1.57%**	**15.23%**	**16.80%**
	Call not made (“no”-call absent)	2052	754	2806
		**60.83%**	**22.35%**	**83.19%**
Total		2105	1268	3373
		**62.40%**	**37.59%**	

The bold values represent a percentage of the total per condition.

**Figure 7:**
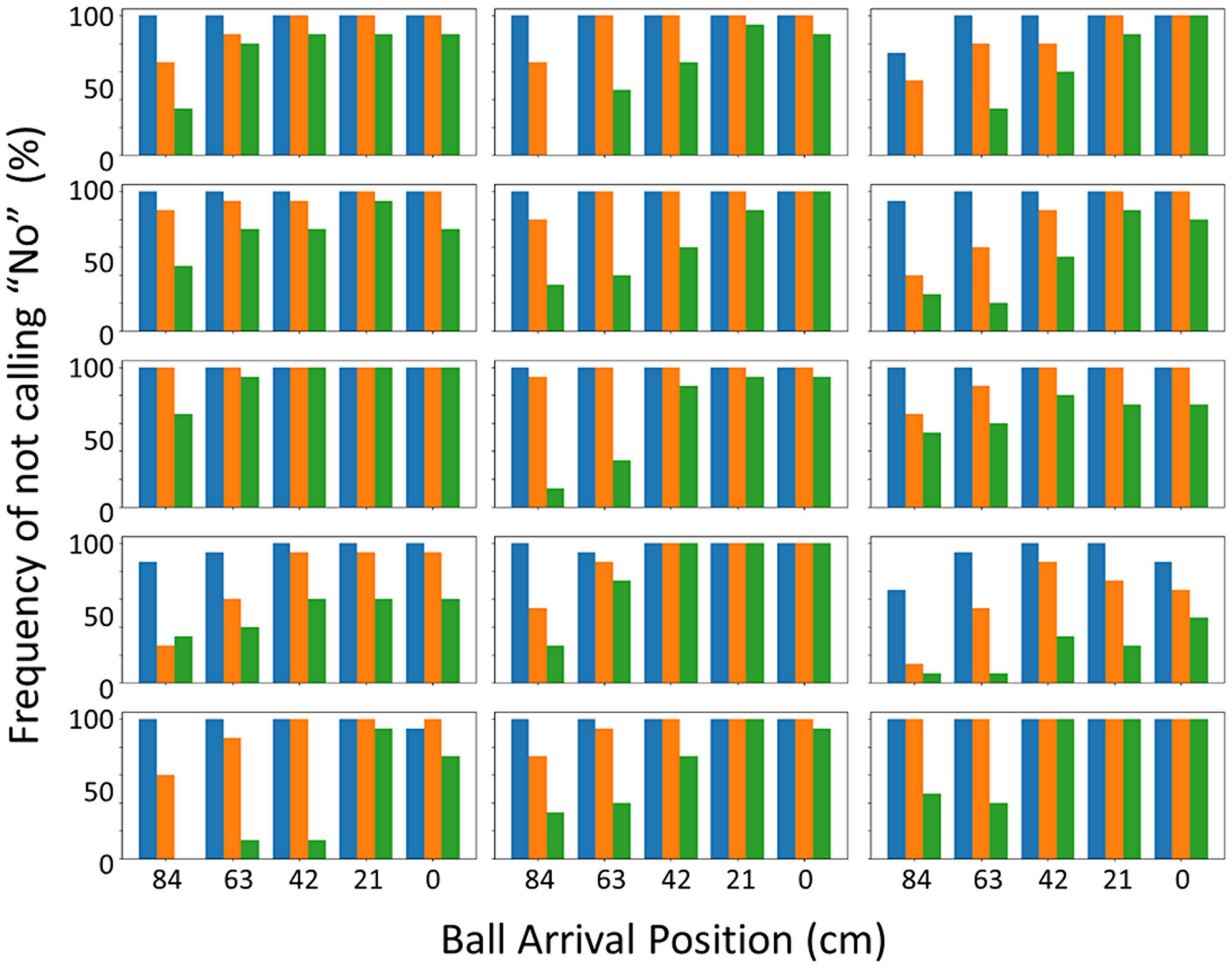
Percentage frequency of not calling “no” in the *judging* session per participant as a function of BAP and ball flight time. The three ball flight times, respectively, were 1.2 s (slow – blue), 0.8 s (intermediate – orange) and 0.6 s (fast – green). The starting position was at the right-most BAP (0 cm).

The main question to be answered is how accurate the “no”-calls were. Before turning to this question, let us first inspect when participants actually gave their “no”-calls. To make it easier to compare this with the success rates of the *action* session in Figure 3 (i.e., actual interceptability), we computed percentages of “no”-s that were not given (i.e., trials in which the participants did not feel that the ball would be uninterceptable). These percentages are presented in Figure 7, as a function of ball arrival position and ball flight time. The two figures paint a similar picture, but also show differences. Overall, Figure 7 shows the same boundary at longer distances from the start especially at higher vertical ball speeds, again with individual differences, as Figure 3 showed for interceptability. However, the effect of a decrease in interception success rate simply as a function of ball flight time, as visible in Figure 3 for the shorter distances from the start, did not seem to be present in the interceptability judgments (Figure 7).

**Figure 8:**
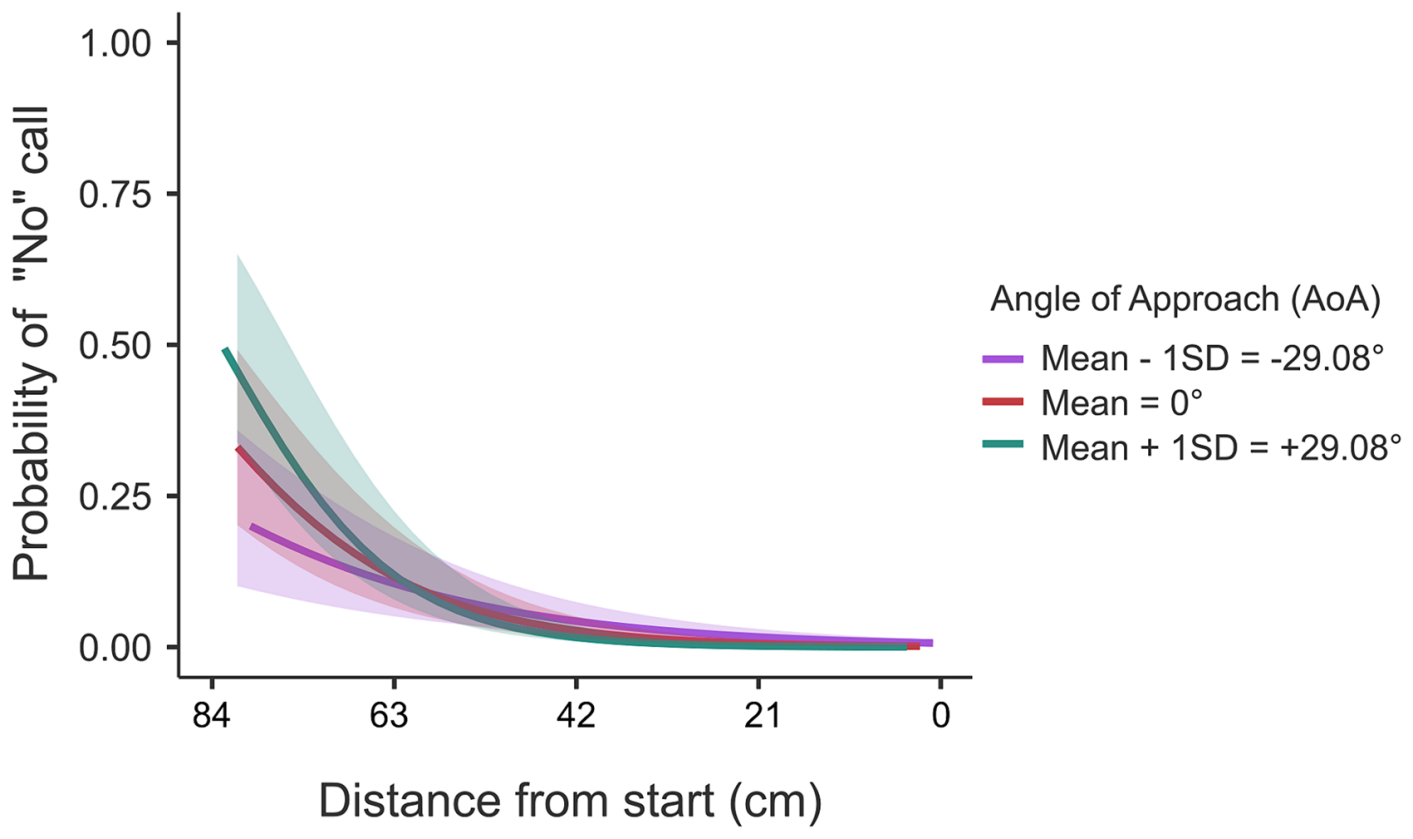
Probability of verbal judgment being made (1 = “no”-call) as a function of distance from start and angle of approach (AoA). A significant interaction effect of distance and AoA is observed.

Taken together, the first inspection of the results seemed to indicate that judgments were related with a boundary that was determined by a combination of ball arrival position and ball flight time. To substantiate this more formally, we built a GLMER model for verbal judgments in similar fashion as we did for the results for interceptability. The final model (Table 2: Verbal Judgment; see also Supplementary Table S3) mirrors^2^ the model for interceptability, with the exception of the D x T interaction effect that was present for interceptability but absent for the judgments. Also, as can be seen in Figure 8, in contrast to the situation for actual interceptability, the effects of the angle of approach for the judgments only played out for the longer distances from the starting location.

Now that we scrutinized the factors that affected the presence of “no”-calls, the next question is how accurate these calls were. To assess this, we used the GLMER model of interceptability from the *action* session. Because of the randomization of the offset of ball departure and arrival position (as detailed in the Methods section, the complete ball trajectory was shifted a random distance to the left or to the right on each individual trial), a direct comparison of judgments and the actions was not possible. However, using the GLMER model from the *action* session (Table 2: Interceptability), we could predict interceptability for individual trials in the *judging* session by simply entering the details of these trials (i.e., their distance from the starting location, ball flight time, angle of approach and participant).

When we compare the presence and absence of “no”-calls with the predictions for the interceptability model, Table 4 shows that there was a congruence of 83.13% between predicted and perceived interceptability. That is to say, summing the percentages of trials with absent “no”-s when the model predicted an interception (68.78%) and the trials in which a “no” was called and the model indicated that the ball was uninterceptable (14.35%), almost 85% of the calls turned out to be accurate. Whereas only few (2.46%) cases showed up in which the ball was predicted to be interceptable but still a “no”-call was made, the situation of not calling “no” while the ball was predicted to be interceptable occurred in almost 15% of the trials.

**Table 4:** Classification table of *judging* session with respect to model predicted interceptability result (interceptable or uninterceptable) and frequency of verbal calls being made (“no”-call present) or not (“no”-call absent).

		Model predicted result		Total
		Interceptable	Uninterceptable	
Verbal Judgment	Call made (“no”-call present)	83	484	567
		**2.46%**	**14.35%**	**16.80%**
	Call not made (“no”-call absent)	2320	486	2806
		**68.78%**	**14.40%**	**83.19%**
Total		2403	970	3373
		**71.24%**	**28.75%**	

The bold values represent a percentage of the total per condition.

Table 2 (Congruency; see also Supplementary Table S4) presents the GLMER model that we built to establish which factors were affecting the congruency between the “no”-s and the predictions from the interceptability model. The same factors turned out to play a role also for congruency. Congruency was less at shorter distances and at longer flight times. This effect, again, seems to reflect the result that ball flight time in itself was not affecting the “no”-calls, perhaps best exemplified with the lack of an effect of these factors on the “no”-s for balls arriving around the paddle starting position (see Figure 8). This difference in how ball flight time affected both the boundary of to where on the interception axis participants were still able to move their paddle, which seemed to show up both for interceptability and its judgments, and how it affected interceptability but not its judgments in general, showed up as the D x T interaction effect in Table 2 (Congruency). Finally, the effects including the angle of approach in Table 2 on Congruency are harder to interpret but are in the same direction as those in Table 2 (Interceptability).

## 4 Discussion

In the present study, we set out to delineate the affordance of interceptability for oneself. To this end, participants performed a lateral interception (*action*) task with a range of interceptable and uninterceptable balls. They also performed a *judging* task, wherein they concurrently made verbal judgments about the perceived interceptability on each trial. We examined the properties of this agent-environment system that varied consistently with the interceptability of a ball for each agent. We found that a combination of the following variables best predicted interceptability for oneself: distance to the interception location, ball flight time and the angle of approach of the ball.

It turned out that ball flight time had two different effects on interceptability. On the one hand, as hypothesized, the ball flight time influenced the boundary between interceptable and uninterceptable balls. This boundary emerged as a function of the distance to be covered in a certain time. Given that in a certain time one could only cover a certain distance, the shortest ball flight times naturally led to lower likelihoods of success for larger distances. This was evident through the significant main effects of ball flight time and distance on the success rate (see Table 2: Interceptability). That is, for the same distance, faster balls led to worse performance and for the same ball flight times, larger distances led to worse performance. On the other hand, ball flight time had a negative effect on accuracy even for balls that were within this boundary. Surprisingly, even for shorter distances, the fastest balls were consistently missed more often than the slower balls. This speed-accuracy trade-off can be seen in the significant interaction effect between the variables of ball flight time and distance on interceptability, particularly close to the paddle starting position, where 95% confidence intervals did not overlap (see Figure 4).

An unanticipated factor that turned out to affect interceptability was the angle under which the ball approached the interception axis. On average, more balls were intercepted when the balls were also moving leftward (like the paddle) than when they were moving rightward, although this effect reversed for balls arriving at the longest distance from the starting location (i.e., near the left of the screen with the starting position located near the right of the screen; see Figure 5). This might be related with the angle-of-approach effect that has been documented in the literature on the visual control of interception (Montagne et al., 1999; Michaels et al., 2006; Arzamarski et al., 2007; Ledouit et al., 2013). The latter effect refers to systematic differences in end-effector kinematics in situations where balls arrive at exactly the same interception location, take the same time to get there, but do so arriving under different approach angles (or, more generally, following different trajectories). One thing that this angle-of-approach effect shows is that the control of interception is continuous rather than based on a prediction of an arrival position from information gathered early on in the movement. Indeed, if the latter were the case, no systematic effects of how the target would reach an interception location would be expected (*cf.* Peper et al., 1994; Montagne et al., 1999; Ledouit et al., 2013). Second, the angle-of-approach effect has been indicative of the nature of the prospective information that is being used in this continuous visual control as it points at a combination of two nulling strategies: a zeroth-order strategy of seeking to null the angle between the line connecting ball and end effector and the vertical (i.e., equivalent to seeking to bring the end effector directly under the moving ball) and a first-order strategy of seeking to bring the same angle to a constant value (i.e., for rectilinear ball trajectories, equivalent to seeking to bring the end effector directly to the future interception location). Indeed, the angle-of-approach effect can only be explained by a combination of both strategies (*cf.* Ledouit et al., 2013; see Bootsma et al., 2016 for an account in which a single time derivative of fractional order would explain this effect). The implication of all this is that for all ball trajectories leading to the same ball arrival position, in the control of interception, on average during the movement, the end effector would be more positioned in the direction from which the ball would be arriving (i.e., more to the right for balls horizontally moving leftward, and vice versa).

**Figure 9:**
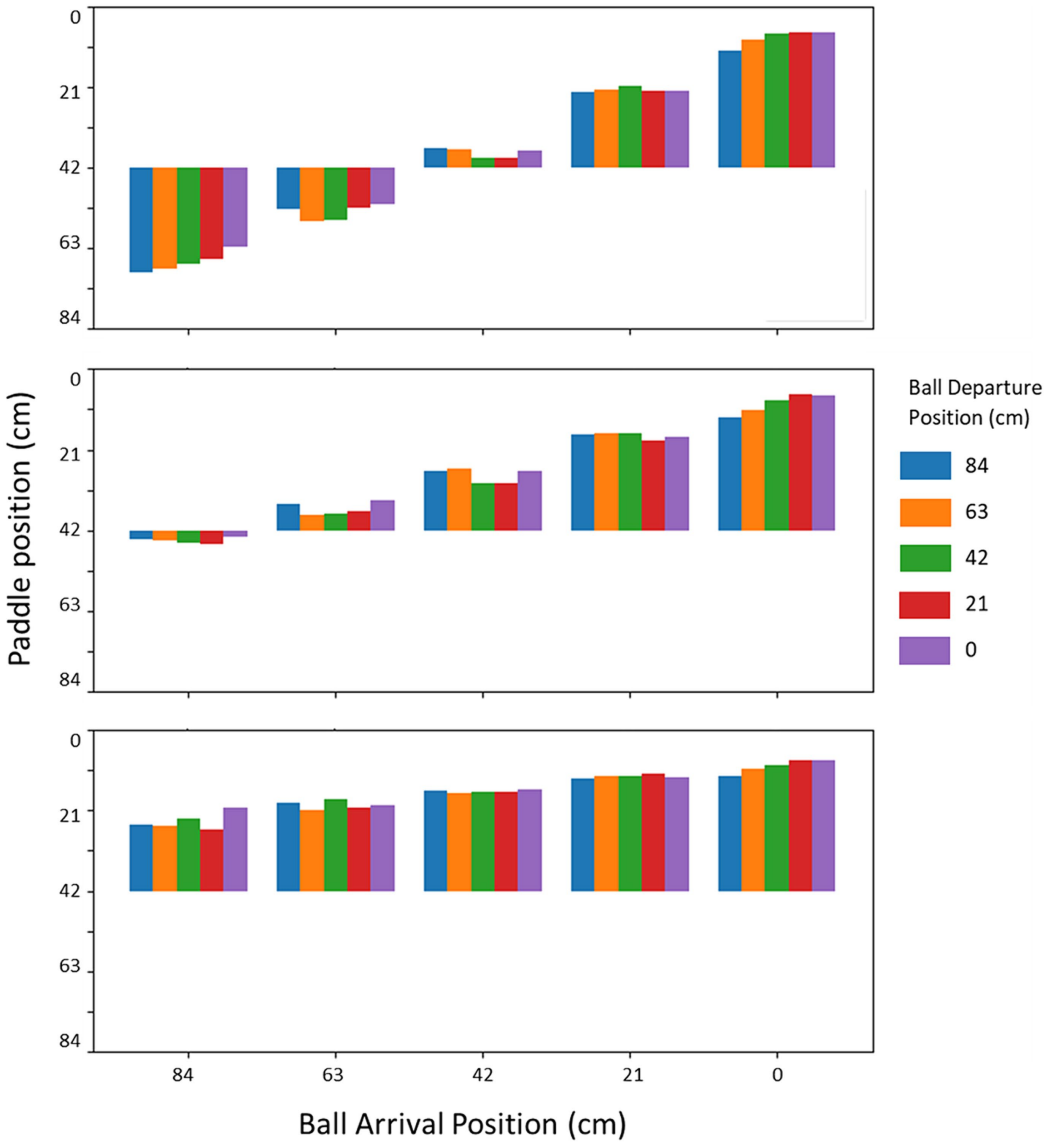
Position of the paddle at one third of the ball flight time before successful interception/passing the interception axis as a function of the ball arrival position for each ball departure position. The topmost panel is for the slowest ball flight time (1.2 s), middle panel for the intermediate ball flight time (0.8 s) and the bottom panel is for the fastest ball flight time (0.6 s).

There was an effect of the angle of approach on interceptability, but this was not reflected in the kinematics. When we inspected the paddle kinematics, it turned out that this typical angle-of-approach effect was not clearly present. Figure 9 gives the average paddle position after two thirds of ball flight time has passed at each of the (binned) ball arrival positions, for each of the (binned) ball departure positions and for each ball flight time. At some ball arrival positions, a hint of the angle-of-approach effect can be seen. For instance, for balls arriving most to the right side of the screen (i.e., around the paddle starting position), on average, the paddle had moved more leftward for balls coming from ball departure positions more toward the left. A similar pattern can be observed for the balls arriving most to the left side of the screen, most notably for the longest ball flight time. However, the effect was certainly not consistent across all ball arrival positions. This may be due to several reasons: Contrary to earlier work, in the current experiment the initial paddle position was always far to the right side of the screen, the experiment used relatively high vertical ball speeds (short ball flight times), and included uninterceptable balls by design, to name a few candidates. However this may be, even without a convincing angle-of-approach effect in the action kinematics themselves, interceptability seemed to include such an effect to some extent.

Also, the lack of an effect of the minimum movement time measure on interceptability was an unexpected result. We established how long it took each individual to cover the full window width with their onscreen paddle and had expected that this measure would be related, at least, with the individual interceptability of balls. For a person who needed less time to move to the other side of the screen, we hypothesized, more balls farther away from the paddle starting location would be reachable, and, thus, would be interceptable. However, we found no effect of this measure of the individuals’ action capability on their ability to intercept a ball. Also, we found no effects of this individual action measure on any of the other variables such as success rate or frequency of calling “no.” In none of our GLMER models, this variable of the time needed to cover the screen width turned up as a significant predictor. The reasons for such lack of an effect of this particular measure on interceptability and its judgments might be manyfold. The obvious candidate is that this time variable simply does not capture the relevant individual action capabilities. Postma et al. (2018) demonstrated that the distance that an individual could cover in a fixed time codetermined the interceptability of approaching fly balls. That is to say, these authors were able to identify a variable that represented the actor side of the affordance of interceptability of fly balls. In a later study, Postma et al. (2022) suggested that both the maximum running speed that someone can attain as well as their maximum running acceleration both contributed to this maximum distance given a certain time-to-interception. The measure that we adopted in the present study seems highly similar to the measures used by Postma and colleagues. Still, this variable could not be demonstrated to play a significant role in the affordance of interceptability in the lateral interception task that we studied here. The lack of finding an effect of minimum movement time could, of course, also be related to the power of our study design. We did indeed not design the study for explicit hypothesis testing but rather as a first step in developing a model that captures interceptability. However, the absence of minimum movement time as a significant predictor in each of the GLMER models that we built indicates that, while this factor may still play a role, it is definitely not the strong predictor that we expected it to be. In future work on this issue, explicit testing of the models that we presented may be designed, with the presented results allowing formal *a priori* determination of sample size.

We not only studied actual interceptability (i.e., what makes a ball interceptable or not?) but also perceived interceptability (what factors determine which balls are judged to be interceptable or not, and how do these judgments compare to the actual interceptability of the balls?). In the *judging* task, participants were confronted with similar (but not identical) situations as in the *action* task, but now they were instructed to call “no” when they felt that a ball would be uninterceptable. This call could be made at any moment during the interception attempt, after which the attempt could be abandoned. This implies that we have two types of result: trials with a “no”-call and trials in which participants did not make a call and continued to try to intercept the ball. Figure 7 gives the rates of the latter situation, to be compared with Figure 3 with interception-success rates in the *action* session. The two figures show similar patterns but also one difference. Whereas interceptability (i.e., in the *action* session) was clearly affected by vertical ball speed *per se* –even for balls heading toward the starting location, a clear effect of ball flight time was present–this was not the case for the interceptability judgments. Our analyses indicated that these judgments were affected by the same factors as was interceptability, but the interaction effect of ball arrival position and ball flight time was absent.

With the two effects of ball flight time (or, vertical ball speed, if you will) on interceptability (i.e., the effect that some distance could not be covered with the available time and the effect that more balls were missed for higher vertical ball speeds) that were not both present in the interceptability judgments, a score of 83% correct (lack of) “no”-calls seems quite good. This percentage was determined by using the GLMER model for interceptability (*action* session) to predict interceptability for all individual trials in the *judging* sessions. We followed this method because the ball trajectories in both sessions were similar but not identical because of the random offset that we applied to shift ball trajectories slightly on each trial. So, in 17% of the trials, the ball was either interceptable but still a “no” was called, or the ball was uninterceptable without any call. Our analyses also indicated that in over 20% of the trials the interception turned out to be unsuccessful while participants had not indicated the ball to be uninterceptable. Apart from the potential reason for the discrepancy mentioned before (i.e., the judgments did not consider the speed-accuracy effects), of course, we cannot rule out the possibility that perceptual judgments simply do not reflect well the capacity of individuals to know action boundaries. A number of studies have suggested this to be the case. For instance, several studies concluded an underestimation of perceived ability (Mark, 1987; Pepping and Li, 2000; Pufall and Dunbar, 1992), whereas another body of work concluded an overestimation of people’s abilities (Warren and Whang, 1987; Carello et al., 1989; Mark et al., 1997). The presence of such a discrepancy may cast doubt on the method of relying on verbal judgments for studying affordance perception (Heft, 1993; Jiang and Mark, 1994; Rochat and Wraga, 1997; Pagano and Bingham, 1998; Pepping and Li, 2005). Still, our results do seem to indicate that people are, to a certain extent, able to know about interceptability in this task.

If we accept that people are able to know about interceptability, the next question would be how they are able to know. What information is available to them to know about interceptability? In the present study, we have not made an attempt to identify this information. One way to approach this issue would be to study the temporal evolution of available optical angles around the moments that our participants gave their “no”-calls. Inspection of the same optical angles during the actual interception, and relating the two, perhaps would even be a way to bridge the gap between affordance perception and the online control of action. That is to say, such an endeavor might contribute to finding common ground between these two aspects of natural behavior, finding common ground to what until now has been contrasted as two types of theorizing regarding the control of action: affordance-based control and current-future control (*cf.* Postma et al., 2018, 2022; Steinmetz et al., 2020; see also: Fajen, 2021).

In conclusion, the present contribution examined the affordance of interceptability for oneself. Our goal was to elucidate the nature of this affordance in terms of critical factors of the agent-environment system that could capture behavior. A second goal was to scrutinize whether people could perceive the interceptability of a ball for themselves, and if so, how reliable their judgments were. We found that firstly, a handful of physical variables could capture interceptability and contribute to an action-boundary between interceptable and uninterceptable balls. Amongst these variables was the spatiotemporal boundary in addition to the angle of approach of the ball. Secondly, we found that there was a dual role of the temporal constraint (ball flight time), which adversely affected the accuracy of participants’ actions. Finally, we established that people could reliably judge interceptability with a high degree of accuracy. All in all, the present contribution provides a stepping stone to developing a complete formulation of interceptability for one person, with the future direction heading toward understanding the informational basis of this affordance.

## Data availability statement

The raw data supporting the conclusions of this article will be made available by the authors, without undue reservation.

## Ethics statement

The studies involving humans were approved by Ethics Board of the UMCG (University Medical Center Groningen, University of Groningen, Netherlands). The studies were conducted in accordance with the local legislation and institutional requirements. The participants provided their written informed consent to participate in this study.

## Author contributions

SD: Conceptualization, Data curation, Formal analysis, Investigation, Methodology, Visualization, Writing – original draft, Writing – review & editing. RB: Conceptualization, Formal analysis, Funding acquisition, Methodology, Project administration, Supervision, Writing – original draft, Writing – review & editing, Validation. FZ: Conceptualization, Funding acquisition, Project administration, Supervision, Validation, Writing – original draft, Writing – review & editing, Formal analysis, Methodology, Visualization, Software.

## Footnotes

^1^In this GLMER model, and the models presented later, we also considered the order in which the *action* and *judging* sessions were administered in each participant as well as the different blocks of trials. Since both factors did not have a significant effect on the success rates, these factors were not retained in the final models.

^2^Note that, because a “no” in the *judging* session corresponds to a miss in the *action* session, opposite signs of the estimates reflect comparable effects.
