# Supplementary material for: Can I catch this ball and do I know if I can? Characterizing the affordance of interceptability for oneself
Source: Front Psychol. 2024 May 30;15:1397476. doi: 10.3389/fpsyg.2024.1397476 (PMC11177208; doi:10.3389/fpsyg.2024.1397476)
Supplement: Supplementary file 1 [file Data_Sheet_1.PDF]

Supplementary Material

Table S1: GLMER analysis on success in *training* session.

Model Info

Info	Value	Comment
Model Type	Logistic	Model for binary y
Call	glm	Result ~ 1 + Block + (1 | Participant)
Link function	Logit	Log of the odd of y=1 over y=0
Direction	P(y=1)/P(y=0)	P(Result = success) / P(Result = miss)
Distribution	Binomial	Dichotomous event distribution of y
LogLikel.	-627.5282	Unconditional Log-Likelihood
-2*LogLikel.	1255.0564	Unconditional absolute deviance
Deviance	1229.2486	Conditional relative deviance
R-squared	0.0398	Marginal
R-squared	0.0888	Conditional
AIC	1263.0600	Less is better
BIC	1285.9312	Less is better
Residual DF	2246.0000	
Chi-squared/DF	0.9441	Overdispersion indicator
Converged	yes	
Optimizer	bobyqa	

Fixed Effect Omnibus tests

	X^2	df	p
Block	26.1	2.00	< .001

Fixed Effects Parameter Estimates

				95% Confidence Interval				
**Names**	**Effect**	**Estimate**	**SE**	**Lower**	**Upper**	**exp(B)**	**z**	**p**
(Intercept)	(Intercept)	2.527	0.138	2.256	2.80	12.52	18.25	< .001
Block-1	2 – 1	0.664	0.181	0.308	1.02	1.94	3.66	< .001
Block-2	3 – 1	0.894	0.193	0.515	1.27	2.44	4.62	< .001

Random Components

Groups	Name	SD	Variance	ICC
Participant	(Intercept)	0.421	0.177	0.0510
Residuals		1.000	1.000	.

Note. Number of Obs: 2250, groups: Participant 15

Post Hoc Comparisons - Block

Comparison						
**Block**		**Block**	**exp(B)**	**SE**	**z**	**p**
1	-	2	0.515	0.0934	-3.66	< .001
1	-	3	0.409	0.0791	-4.62	< .001
2	-	3	0.794	0.1699	-1.08	0.282

Table S2: GLMER analysis on Interceptability in *action* session. Abbreviations: D: distance from start, T: ball flight time comparisons at two levels (T1: 0.8 s - 0.6 s and T2: 1.2 s - 0.6 s), and AoA: angle of approach.

Model Info

Info	Value	Comment
Model Type	Logistic	Model for binary y
Call	glm	Interceptability ~ 1 + D + T + AoA + D x T + D x AoA + (1 | Participant)
Link function	Logit	Log of the odd of y=1 over y=0
Direction	P(y=1)/P(y=0)	P(Result = success) / P(Result = miss)
Distribution	Binomial	Dichotomous event distribution of y
LogLikel.	-1606.643	Unconditional Log-Likelihood
-2*LogLikel.	3213.287	Unconditional absolute deviance
Deviance	3170.924	Conditional relative deviance
R-squared	0.381	Marginal
R-squared	0.413	Conditional
AIC	3231.290	Less is better
BIC	3286.404	Less is better
Residual DF	3366.000	
Chi-squared/DF	0.972	Overdispersion indicator
Converged	yes	
Optimizer	bobyqa	

Fixed Effect Omnibus tests

	X^2	df	p
D	97.6	1.00	< .001
T	583.0	2.00	< .001
AoA	22.5	1.00	< .001
D ✻ T	28.7	2.00	< .001
D ✻ AoA	59.8	1.00	< .001

Fixed Effects Parameter Estimates

				95% Confidence Interval				
Names	Effect	Estimate	SE	Lower	Upper	exp(B)	z	p
(Intercept)	(Intercept)	1.230	0.1259	0.9837	1.477	3.423	9.77	< .001
D	D	-0.664	0.0672	-0.7957	-0.532	0.515	-9.88	< .001

Fixed Effects Parameter Estimates

Names	Effect	Estimate	SE	95% Confidence Interval Lower	95% Confidence Interval Upper	exp(B)	z	p
T1	0.8 - 0.6	1.398	0.0978	1.2059	1.589	4.046	14.29	< .001
T2	1.2 - 0.6	3.147	0.1340	2.8843	3.410	23.268	23.48	< .001
AoA	AoA	0.293	0.0618	0.1718	0.414	1.340	4.74	< .001
D ✻ T1	D ✻ 0.8 - 0.6	0.258	0.0999	0.0625	0.454	1.295	2.59	0.010
D ✻ T2	D ✻ 1.2 - 0.6	0.679	0.1271	0.4302	0.928	1.972	5.35	< .001
D ✻ AoA	D ✻ AoA	-0.392	0.0508	-0.4918	-0.293	0.675	-7.73	< .001

Random Components

Groups	Name	SD	Variance	Variance 95% C.I. Lower	Variance 95% C.I. Upper	ICC
P	(Intercept)	0.421	0.177	0.0794	0.438	0.0511
Residuals		1.000	1.000	.	.	.

Note. Number of Obs: 3375, groups: Participant 15

Table S3: GLMER analysis on Verbal Judgments in *judging* session. Abbreviations: D: distance from start, T: ball flight time comparisons at two levels (T1: 0.8 s - 0.6 s and T2: 1.2 s - 0.6 s) and AoA: angle of approach.

Model Info

Info	Value	Comment
Model Type	Logistic	Model for binary y
Call	glm	Verbal Judg. ~ 1 + D + T + AoA + D x AoA + (1 | Participant)
Link function	Logit	Log of the odd of y=1 over y=0
Direction	P(y=1)/P(y=0)	P(Verbal Judg. = present) / P(Verbal Judg. = absent)
Distribution	Binomial	Dichotomous event distribution of y
LogLikel.	-852.146	Unconditional Log-Likelihood
-2*LogLikel.	1704.293	Unconditional absolute deviance
Deviance	1643.454	Conditional relative deviance
R-squared	0.585	Marginal
R-squared	0.704	Conditional
AIC	1718.290	Less is better
BIC	1761.158	Less is better
Residual DF	3366.000	
Chi-squared/DF	0.724	Overdispersion indicator
Converged	yes	
Optimizer	bobyqa	

Fixed Effect Omnibus tests

	X^2	df	p
D	259.7	1.00	< .001
T	403.3	2.00	< .001
AoA	30.4	1.00	< .001
D ✻ AoA	72.9	1.00	< .001

Fixed Effects Parameter Estimates

				95% Confidence Interval				
Names	Effect	Estimate	SE	Lower	Upper	exp(B)	z	p
(Intercept)	(Intercept)	-3.688	0.3327	-4.340	-3.035	0.02503	-11.08	< .001
D	D	1.700	0.1055	1.493	1.907	5.47386	16.12	< .001

Fixed Effects Parameter Estimates

				95% Confidence Interval				
Names	Effect	Estimate	SE	Lower	Upper	exp(B)	z	p
T1	0.8 - 0.6	-2.360	0.1521	-2.658	-2.062	0.09441	-15.51	< .001
T2	1.2 - 0.6	-4.981	0.2917	-5.553	-4.410	0.00686	-17.07	< .001
AoA	AoA	-0.513	0.0930	-0.695	-0.330	0.59889	-5.51	< .001
D ✻ AoA	D ✻ AoA	0.635	0.0744	0.490	0.781	1.88771	8.54	< .001

Random Components

Groups	Name	SD	Variance	ICC
Participant	(Intercept)	1.15	1.33	0.288
Residuals		1.00	1.00	.

Note. Number of Obs: 3373, groups: Participant 15

Table S4: GLMER analysis on Congruency of verbal judgments and model predictions. Abbreviations: D: distance from start, T: ball flight time comparisons at two levels (T1: 0.8 s - 0.6 s and T2: 1.2 s - 0.6 s) and AoA: angle of approach.

Model Info

Info	Value	Comment
Model Type	Logistic	Model for binary y
Call	glm	Congruency ~ 1 + D + T + AoA + D x T + D x AoA + (1 | Participant)
Link function	Logit	Log of the odd of y=1 over y=0
Direction	P(y=1)/P(y=0)	P(Congruency = 1) / P(Congruency = 0)
Distribution	Binomial	Dichotomous event distribution of y
LogLikel.	-1117.611	Unconditional Log-Likelihood
-2*LogLikel.	2235.223	Unconditional absolute deviance
Deviance	2193.274	Conditional relative deviance
R-squared	0.530	Marginal
R-squared	0.564	Conditional
AIC	2253.220	Less is better
BIC	2308.335	Less is better
Residual DF	3364.000	
Chi-squared/DF	0.942	Overdispersion indicator
Converged	yes	
Optimizer	bobyqa	

Fixed Effect Omnibus tests

	X^2	df	p
D	104.2	1.00	< .001
T	221.6	2.00	< .001
AoA	45.3	1.00	< .001
D ✻ T	89.5	2.00	< .001
D ✻ AoA	11.8	1.00	< .001

Fixed Effects Parameter Estimates

				95% Confidence Interval				
Names	Effect	Estimate	SE	Lower	Upper	exp(B)	z	p
(Intercept)	(Intercept)	2.811	0.1887	2.441	3.1813	16.634	14.90	< .001
D	D	-1.246	0.1221	-1.485	-1.0069	0.288	-10.21	< .001

Fixed Effects Parameter Estimates

				95% Confidence Interval				
Names	Effect	Estimate	SE	Lower	Upper	exp(B)	z	p
T1	0.8 - 0.6	1.619	0.1492	1.326	1.9110	5.046	10.85	< .001
T2	1.2 - 0.6	4.022	0.3601	3.316	4.7279	55.825	11.17	< .001
AoA	AoA	0.520	0.0773	0.369	0.6714	1.682	6.73	< .001
D ✻ T1	D ✻ 0.8 - 0.6	-1.280	0.1376	-1.550	-1.0104	0.278	-9.30	< .001
D ✻ T2	D ✻ 1.2 - 0.6	-0.825	0.3017	-1.416	-0.2337	0.438	-2.73	0.006
D ✻ AoA	D ✻ AoA	-0.210	0.0612	-0.330	-0.0903	0.810	-3.44	< .001

Random Components

				Variance 95% C.I.		
Groups	Name	SD	Variance	Lower	Upper	ICC
Participant	(Intercept)	0.505	0.255	0.115	0.626	0.0719
Residuals		1.000	1.000	.	.	.

Note. Number of Obs: 3373, groups: Participant 15
